# MAPPI-DAT: data management and analysis for protein–protein interaction data from the high-throughput MAPPIT cell microarray platform

**DOI:** 10.1093/bioinformatics/btx014

**Published:** 2017-01-17

**Authors:** Surya Gupta, Veronic De Puysseleyr, José Van der Heyden, Davy Maddelein, Irma Lemmens, Sam Lievens, Sven Degroeve, Jan Tavernier, Lennart Martens

**Affiliations:** 1 Medical Biotechnology Center, VIB, Ghent, Belgium; 2 Department of Biochemistry, Ghent University, Ghent, Belgium; 3 Bioinformatics Institute Ghent, Ghent University, Ghent, Belgium

## Abstract

**Summary:**

Protein-protein interaction (PPI) studies have dramatically expanded our knowledge about cellular behaviour and development in different conditions. A multitude of high-throughput PPI techniques have been developed to achieve proteome-scale coverage for PPI studies, including the microarray based Mammalian Protein-Protein Interaction Trap (MAPPIT) system. Because such high-throughput techniques typically report thousands of interactions, managing and analysing the large amounts of acquired data is a challenge. We have therefore built the MAPPIT cell microArray Protein Protein Interaction-Data management & Analysis Tool (MAPPI-DAT) as an automated data management and analysis tool for MAPPIT cell microarray experiments. MAPPI-DAT stores the experimental data and metadata in a systematic and structured way, automates data analysis and interpretation, and enables the meta-analysis of MAPPIT cell microarray data across all stored experiments.

**Availability and Implementation:**

MAPPI-DAT is developed in Python, using R for data analysis and MySQL as data management system. MAPPI-DAT is cross-platform and can be ran on Microsoft Windows, Linux and OS X/macOS. The source code and a Microsoft Windows executable are freely available under the permissive Apache2 open source license at https://github.com/compomics/MAPPI-DAT.

**Supplementary information:**

Supplementary data are available at *Bioinformatics* online.

## 1 Introduction

The identification and quantification of Protein–Protein Interactions (PPIs) can help to understand cellular responses in health and disease. Despite much effort devoted to the study of PPIs, we still lack complete knowledge of all interacting proteins (Gavin *et al.*, 2011). A variety of methods have therefore been developed to provide a more complete coverage of PPI networks. One of these is the Mammalian Protein-Protein Interaction Trap (MAPPIT) (Lievens *et al.*, 2011) which enables determining interacting partners of proteins in mammalian cells. In MAPPIT, the interaction of two proteins (bait and prey) restores a deficient JAK-STAT signalling pathway. Because activation of the JAK-STAT pathway is also contingent on ligand stimulation, successful expression of the JAK-STAT controlled reporter gene requires both stimulation as well as bait and prey protein interaction. Therefore, non-stimulated replicates are used as control, in which the JAK-STAT stimulus is not provided, and the reporter genes are expected to remain silent. Detailed information about the dataset is provided in Supplementary Material S1. To allow screening of thousands of interactors simultaneously, MAPPIT has been parallelized in the high-throughput based MAPPIT cell microarray system where PPIs are measured by the expression of a fluorescent reporter gene. In its current version up to 17 000 human prey proteins are screened in parallel (Lievens *et al.*, 2016)

Even though the PPI field has benefited greatly from high-throughput approaches like MAPPIT cell microarray, new challenges emerge because of the need to handle the large amounts of data generated. Moreover, accurate statistical data processing is required due to the considerable number of false positive signals that is routinely observed. As a result, it is very important to have access to all data (positive as well as weak or negative interactions) in order to calculate these statistics, or to repeat these calculations at a future time for verification. As such, long-term, structured storage of data along with high quality annotation is essential to ensure the longevity of all data. Interestingly, once such a data storage system is in place, it can also enable meta-analysis across experiments (for instance, to improve statistical power), or allow selected data to be exported to a third-party tool for downstream analysis.

In order to provide the MAPPIT cell microarray system with such a data management and analysis tool, we built the MAPPIT cell microArray Protein Protein Interaction-Data management and Analysis Tool (MAPPI-DAT) as an automated high-throughput data analysis pipeline, capable of processing and storing the thousands of data points acquired in each experiment. MAPPI-DAT is built around a full-featured graphical user interface (GUI) in order to make the tool easy to use for the researcher.

## 2 Tool description and functionality

**Fig. 1.**
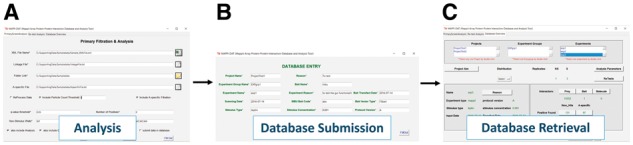
MAPPI-DAT user interface: (**A**) Analysis panel, (**B**) database submission panel and (**C**) database retrieval panel

The MAPPI-DAT user interface consists of three main modules: data analysis, data submission and data retrieval (Fig. 1). The analysis system of MAPPI-DAT comprises three steps: normalization, statistical analysis and post filtration. The MAPPI-DAT data management is built on a relational database back-end.

The fluorescence intensity (integral intensity) measured by the scanner is used as the basis for the analysis of the raw data. The choice for this parameter is explained in Supplementary Material S2. In the analysis, the non-stimulated sample is used as control and the stimulated sample as the test replicates. Detailed information about the analysis can be found in Supplementary Material S3. To account for experiment-wide systematic effects such as plate and within-plate effects, data were normalized using a model described by Wolfinger et al. (2001). The residuals that represent normalized values are then used for further analysis (Supplementary Material S4) using a robust rank-based approach to determine positive interactors (Supplementary Material S3). There are also two post-filtration steps that account for special cases in the data: quartile based filtration and particle count based filtration. A detailed illustration of this filtration is given in Supplementary Materials S5 and S6, respectively.

To ensure the longevity and high-quality annotation of the acquired MAPPIT cell microarray data, MAPPI-DAT can store these data in a relational database through a GUI implemented using the Tkinter Python package. The database is structured to facilitate the storage of raw data, experimental metadata, the analyzed data and all parameter settings (see Supplementary Material S7 for the relational schema). The database retrieval panel in the MAPPI-DAT user interface provides access to detailed information about all existing projects in the database, along with their raw data and analysis results. Moreover, raw data and results can also be downloaded from MAPPI-DAT as tab-delimited text files that can be loaded directly into Cytoscape or R for any desired downstream analyses (see also user manual for some examples). Together with tab-delimited files, user can also download the results in PSI-MI XML format. Finally, these same text files can be imported into another MAPPI-DAT system, where they can be re-analyzed and re-submitted, potentially using different parameter settings and annotations. This in effect allows MAPPI-DAT to function as a data sharing and dissemination platform, where entire datasets can be made available to other interested researchers for analysis and interrogation through MAPPI-DAT.

## 3 Conclusion

We have developed MAPPI-DAT, an automated data storage and analysis system for MAPPIT cell microarray data. MAPPI-DAT facilitates the reduction of the large amount of MAPPIT cell microarray data to a set of true positive interactions, and moreover, through the built-in storage system, it also allows meta-analyses or re-analyses to take place, for instance for advanced pattern mining and knowledge extraction by comparing different experiments. MAPPI-DAT is cross-platform freely available under the permissive Apache2 open source license.

## Supplementary Material

Supplementary Data
