# Supplementary material for: MAPPI-DAT: data management and analysis for protein–protein interaction data from the high-throughput MAPPIT cell microarray platform
Source: Bioinformatics. 2017 Jan 17;33(9):1424–5. doi: 10.1093/bioinformatics/btx014 (PMC5408788; doi:10.1093/bioinformatics/btx014)
Supplement: Supplementary Data [file btx014_supp.docx]

## **Supplement data for**

MAPPI-DAT: data management and analysis for protein-protein interaction data from the high-throughput MAPPIT cell microarray platform

Surya Gupta^1,2,3^, Veronic De Puysseleyr^1,2^, Davy Maddelein^1,2,3^, José Van der Heyden^1,2^, Irma Lemmens^1,2^, Sam Lievens^1,2^#, Sven Degroeve^1,2,3^,Jan Tavernier^1,2*^, Lennart Martens^1,2,3*^

*^1^Medical Biotechnology Center, VIB, Ghent, Belgium, ^2^ Department of Biochemistry, Ghent University, Ghent, Belgium, ^3^ Bioinformatics Institute Ghent, Ghent University, Ghent, Belgium*

*To whom correspondence should be addressed.

Current address: ^#^Orionis Biosciences, Ghent, Belgium

## **S1: MAPPIT cell microarray Dataset structure:**

Mammalian Protein-Protein Interaction Trap (MAPPIT)(Lievens *et al.*, 2011, 2009, 2012; Lemmens *et al.*, 2010), enables determining interacting partners of proteins in mammalian cells. To allow screening of thousands of interactors simultaneously, MAPPIT has been parallelized in the high-throughput based MAPPIT cell microarray system where the interaction of two proteins is determined by activation of a fluorescent reporter gene(Lievens *et al.*, 2016). This is similar to a microarray experiment where fluorescence intensity is used to determine differentially expressed genes (Figure S1 shows an example microscopic image of a MAPPIT cell microarray plate). The fluorescence intensities are measured by an automated scanning microscope, and reported as different types of quantification parameters e.g. cell count, area fraction, and total intensity in a defined area of interest (Supplement S2). MAPPI-DAT primarily relies on the integral intensity parameter for analysis, and the choice for this parameter is explained in Supplement S2. Every data set contains two types of replicates: stimulated replicates, where the JAK-STAT pathway activating stimulus is provided, allowing the interaction between bait and prey to result in reporter gene expression and thus fluorescence; and non-stimulated replicates, in which the JAK-STAT stimulus is not provided, and the reporter genes is expected to remain silent. The non-stimulated replicates thus behave as controls.


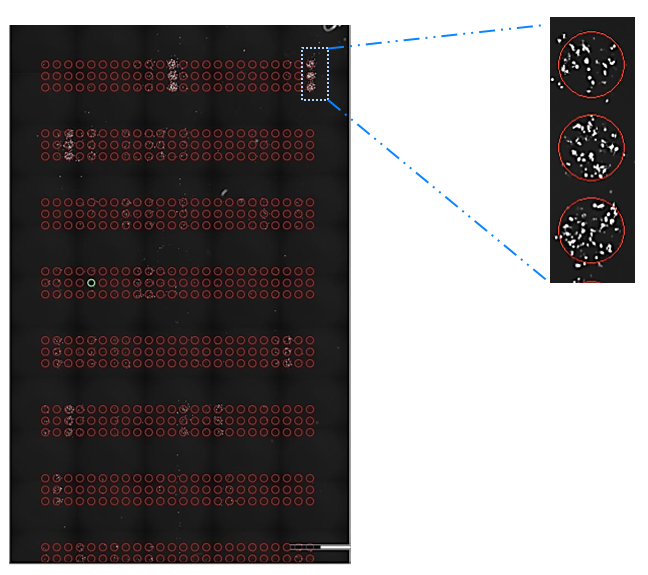


Figure S1 microscopic image of a MAPPIT cell microarray plate. Red circles represent ROIs(regions of interest) and white spots in thesecircles represent fluoroscence intensities. Fluorescence signals are indicative of protein interaction.Empty wells without white spots, and thus without fluorescence,imply no detected proteininteraction.

## **S2-Spearman correlation plot for seven quantification parameters:**

In a MAPPIT cell microarray screen, the acquired raw microscopy data is transformed into seven quantification parameters (see Table S2).

| **Particle Count** | Number of observed particle or group of particle; threshold dependent. |
| --- | --- |
| **Mean Area um^2^** | Region of interest divided by total particle count; threshold dependent. |
| **Grey Value Mean (GVM)** | Total Grey value of all pixels divided by total number of pixels; threshold independent. |
| **Mean Grey Value Mean** | Total of GVM of all particles divided by total particle count; threshold dependent. |
| **Mean Integral Intensity** | Total of GV of all particles divided by total particle count; threshold dependent. |
| **Area Fraction** | Percentage of region of interest; threshold dependent. |
| **Integral Intensity um^2^** | Sum of Grey Values of region of interest divided by total surface of region of interest in µm²; threshold independent. |

Table S2 Detailed information about the seven quantification parameters

Fig S2provides correlation plots with the Spearman correlation coefficient between these seven quantification parameters. It is clear that there are two internally correlated groups of parameters: the first group (marked in blue) includes five quantification parameters, while the second group (marked in green) contains the remaining two quantification parameters. The parameters in the first group (blue) are all dependent on a user defined minimal intensity value threshold which resets all values below that threshold to zero, while the parameters in the second group(green) are both independent of any user-defined threshold.

We have chosen the IntegralIntensity parameter, which is the fluorescence intensity for the region of interest (well), from the second group as the main metric to base analysis on in MAPPI-DAT due to its independence of any user-defined thresholds (Table S2). Note that the GreyValueMean and IntegralIntensity parameters are perfectly correlated and that these are essentially interchangeable (Figure S2). The threshold independence of the integral intensity parameter serves to eliminate variation caused by user differences in defining a threshold, and second, eliminates any problems related to the handling of zeroes during calculations.


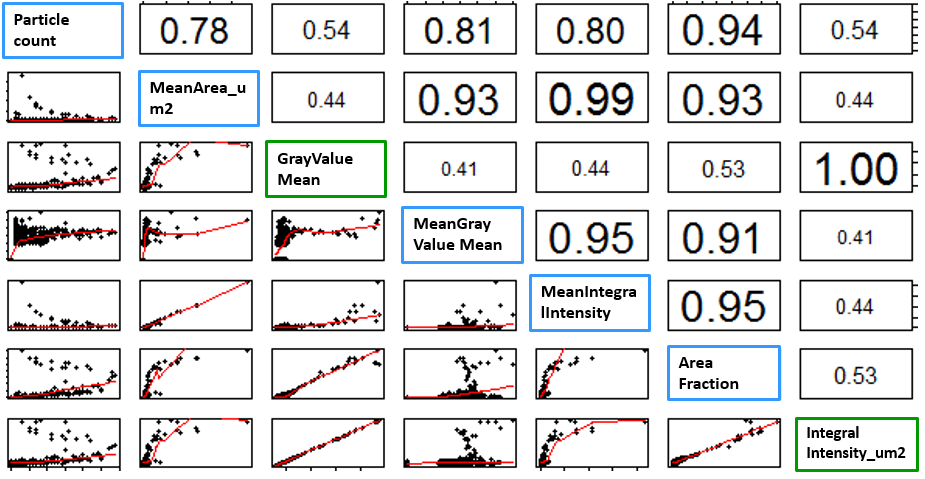


Figure S2 Correlation plot between seven quantification parameter generated from MAPPIT cell microarray screens, with Spearman’s correlation coefficient.

## **S3: Overall MAPPI-DAT analysis workflow**

The analysis system of MAPPI-DAT comprises three steps: normalization, statistical analysis, and post filtration (Figure S3) which are discussed in detail below.

##
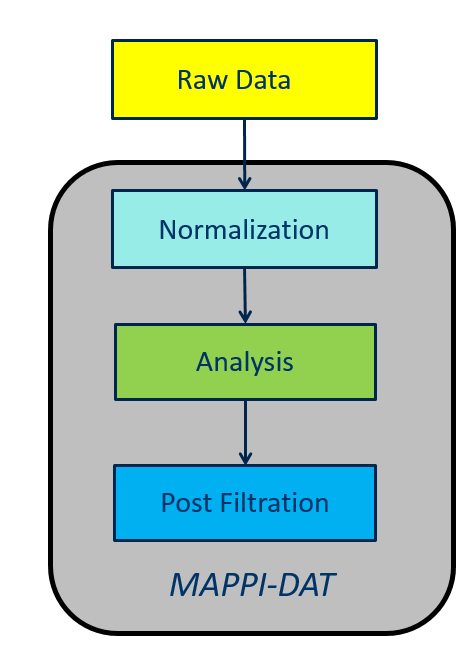


## **Normalization, statistical analysis and post-filtration:**

The fluorescence intensity (integral intensity) measured by the scanner is used as the basis for the analysis of the raw data. The choice of the parameter is explained in Supplement S2. In the analysis, the non-stimulated sample is used as control and the stimulated sample as the test replicates. Before analysis, the integral intensity is first log transformed with base 2, to ensure more constant variation across the intensity range. Then, to account for experiment-wide systematic effects such as plate and within-plate effects, data were normalized using a model described by Wolfinger *et al.*, 2001. The residuals that represent normalized values are then used for further analysis (see Supplement S4 below).

In order to avoid reliance on a normal distribution, a robust rank-based approach (Breitling *et al.*, 2004) is used for the statistical analysis to determine positive interactors. First the fold changes for each of the stimulated data points over the respective non-stimulated data points is calculated. These fold changes are then ranked per replicate experiment; for example, rank rip is the position of protein p in the list of proteins in the ith replicate which is sorted by decreasing fold change so that r=1 for the most protein pair giving the strongest signal. The product of these ranks across replicates is then used to calculate a p-value according to Heskes et al., which is specifically designed to calculate accurate p-values across replicate experiments. In order to cope with the multiple testing problem, the final selection was based on FDR-adjusted p-values using the q-value package in R (Storey, 2002).

Figure S3 Three-step MAPPI-DAT analysis workflow to filter real positives from raw data.

There are also two post-filtration steps that account for special cases in the data. In the first of these steps, interactors with high non-stimulated fluorescence intensities are handled. Indeed, even though a high intensity should represent an interaction between the bait and prey protein, a high intensity in non-stimulated replicates signals auto-activation where the prey protein activates the reporter genes by itself. Therefore, quartile filtration is applied to all non-stimulated replicates to detect fluorescence intensities that are significantly above background level. This filtration uses the commonly applied threshold of 1.5 times inter-quartile range (IQRs) above third quartile. If a particular protein pair then shows significant fluorescence intensity in more than half of the non-stimulated replicates, the interaction is tagged as a false positive. A detailed illustration of this filtration is given in Supplement S5 below. In the second post-filtration step, interactors with significant fold changes between stimulated and non-stimulated samples, but with low stimulated fluorescence intensities are addressed. In these cases, the particle-count parameter, which measures the number of illuminated cells (or clusters of cells) recorded in the microscopic image, is used for filtering (Supplement S2). The particle-count filter can handle two types of cases: first, if the median particle-count in stimulated replicates is lower than the median particle-count in the non-stimulated replicates, and second, if the particle-count in more than half of the stimulated replicate is less than a minimal particle-count defined by the user. A detailed illustration of this filtration is given in Supplement S6 below.

## **S4- effect of normalization on the integral intensity parameter**

Fig S4showsthe effect of normalization on the integral intensity parameter. A single experiment, comprising nine plates is shown. Each plate comes with a replicate, as shown by the colour code, yielding a total of eighteen plates


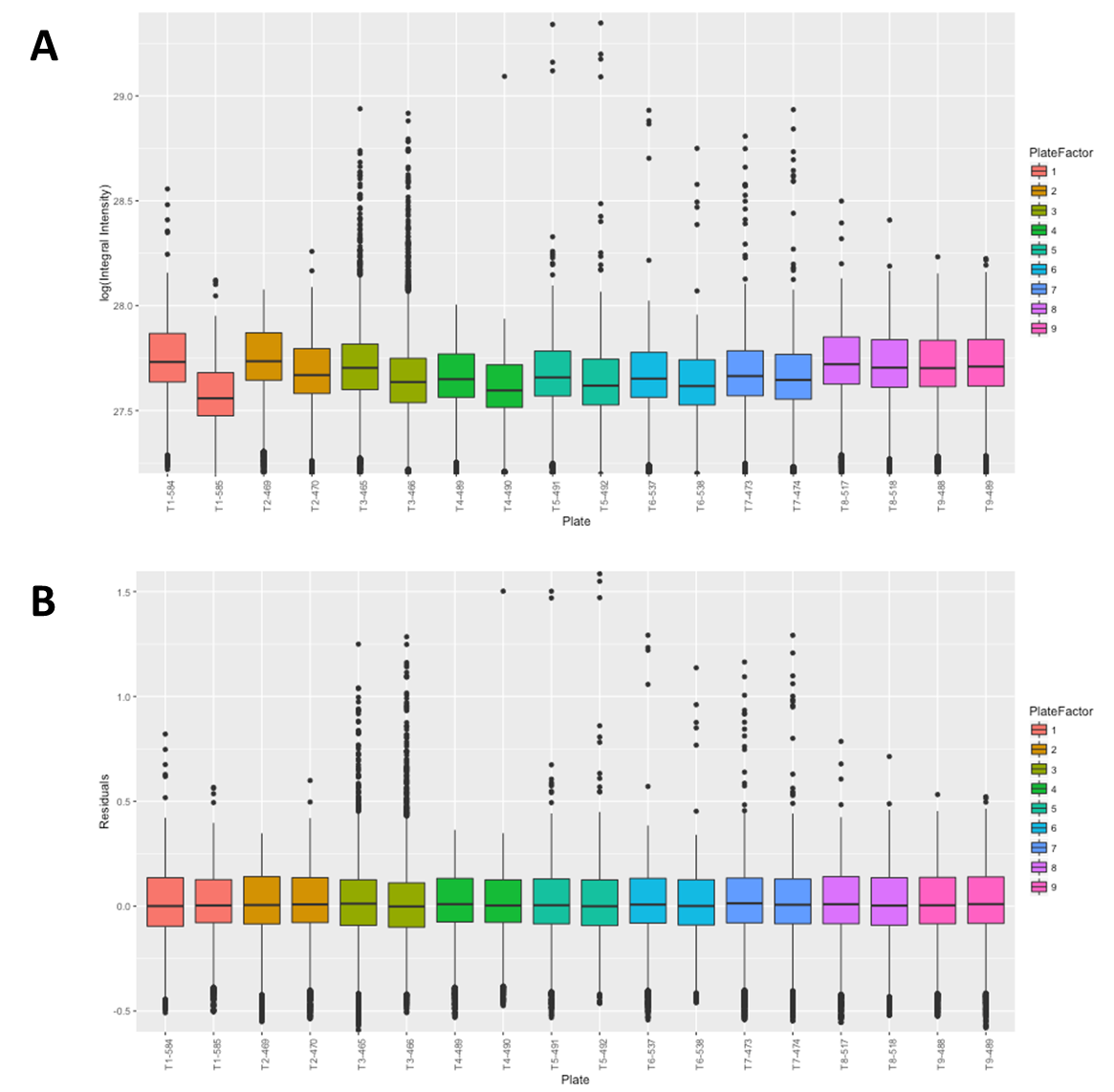


Figure S4A) box plots of the original integral intensity for each plate, replicates are indicated by colour code. B)box plots of the normalized residuals of the integral intensity after ANOVA for each plate, replicates are indicated by colour code.

## **S5-Quartile based filtration:**

In Fig S5, (A) shows histogram distribution of stimulated(blue) and non-stimulated (orange) replicates with significant q-value and fold change showing the change between the non-stimulated and stimulated replicate is significant across all replicates. For this particular case, the stimulated replicate intensity is high which is also supported by the microscopic image (as shown in the black box), however, the non-stimulated intensity is also higher than expected, also shown in the corresponding microscopic image. This shows that it is a case of auto-activating prey where the prey fusion protein can activate the pathway by itself. But these cases cannot be accounted with the analysis method, as analysis only considers the change in the intensity between two conditions which is high in this case (1.39-fold change). However, in normal MAPPIT experiments, positives should not show activation in the non-stimulated replicates. Therefore, to consider these cases we decided to use Quartile based filtration which create a flexible yet effective separation between the background and interaction intensity for the non-stimulated replicates as shown in the green colour in (B). After applying this Quartile based filtration in non-stimulated replicates we were able to spot these cases as false positives as shown in the (B).


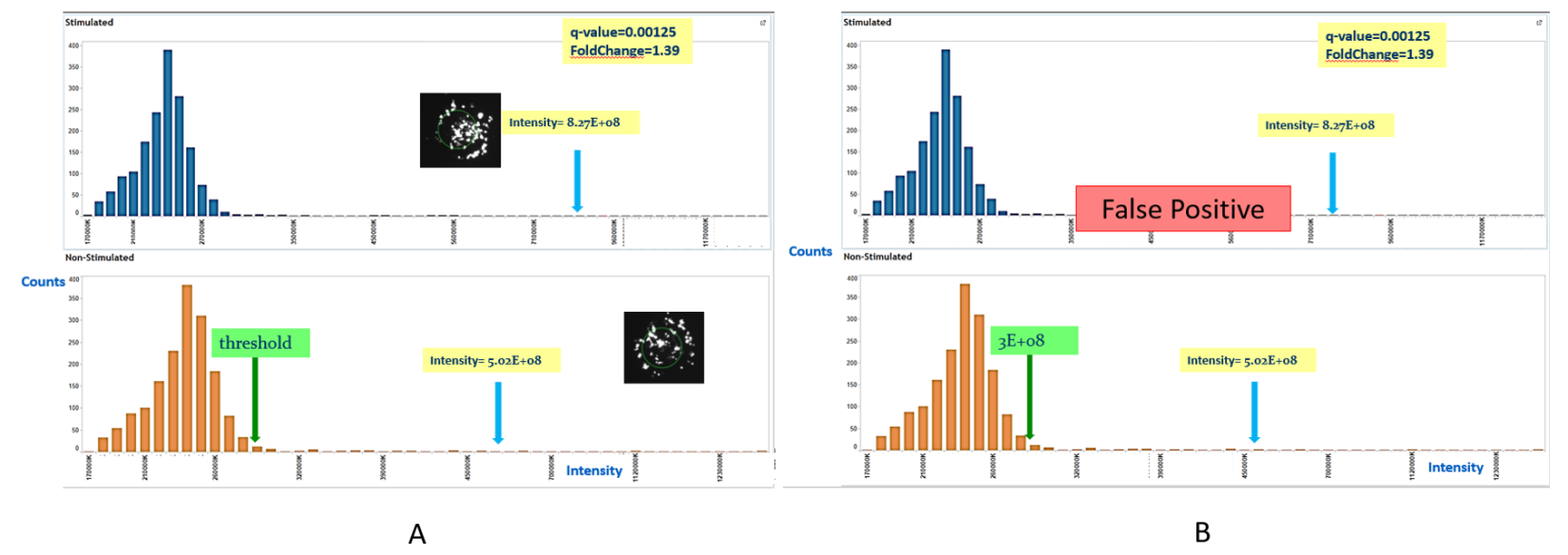


Figure S5 The above figure shows an example case where quartile based filtration is applied. Blue histogram shows distribution for stimulated replicate, and orange histogram shows distribution for non-stimulated replicate. Black boxes show microscopic images of fluorescence intensities in ROIs for corresponding intensities.

## **S6- Particle-count based filtration:**

There are few cases in the MAPPIT cell microarray experiments, where, interactors with significant fold changes between stimulated and non-stimulated samples, lacks sufficient stimulated fluorescence intensities. As shown in Figure S6 (A), where blue colour histogram shows stimulated replicate and orange colour histogram shows non-stimulated replicate, for most of the replicate, integral intensity for the stimulated replicates are higher than the non-stimulated replicate but closer to the background intensity. These cases are not non-significant as there are significant change from the non-stimulated to the stimulated replicate and might be interested for the treatment based experiments, where we visualize the effect on interaction based on type of treatment. But these cases get less priority in non-treatment based experiments, therefore it is important to label these cases. To address this, we have used particle-count based filtration. The particle-count is one of the seven quantification parameter which falls in first parameter group, as explained under section S3. Particle-count parameter, measures the number of illuminated cells (or cluster of cells) recorded in the microscopy image. With particle-count based filtration, we can spot two types of cases: first, if the median particle-count in stimulated replicates is lower than the median particle-count in the non-stimulated replicates, and second, if the particle-count in more than half of the stimulated replicate is less than a minimal particle-count defined by the user.


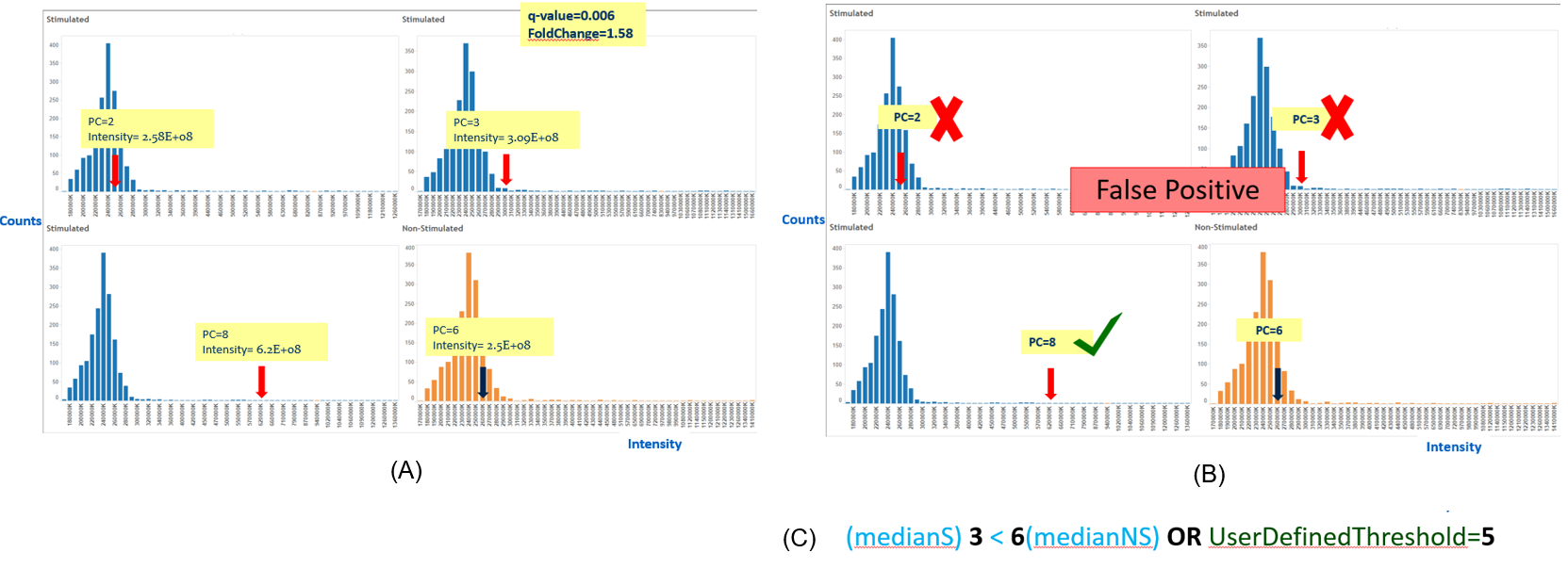


Figure S6 The above figure shows an example of the case where we applied particle based filtration. Blue histogram shows distribution for stimulated replicate, and orange histogram shows distribution for non-stimulated replicate. Black boxes show microscopic images of fluorescence intensities in wells for corresponding intensities.(C) shows an example of two conditions in particle count filtration.

As shown in (C), the median particle-count of the stimulated replicates is lower than median particle-count of non-stimulated replicates. As indicated with the green and red arrows in (B), two out three replicates also have particle count smaller than the user defined threshold (C). With these two types of checks, we can easily label those cases in the MAPPIT cell microarray experiments which have lower stimulated intensity than expected, using user defined threshold and have on an average lower signal than the non-stimulated replicate using median based comparison.

## **S7- Database schema for MAPPI-DAT system**


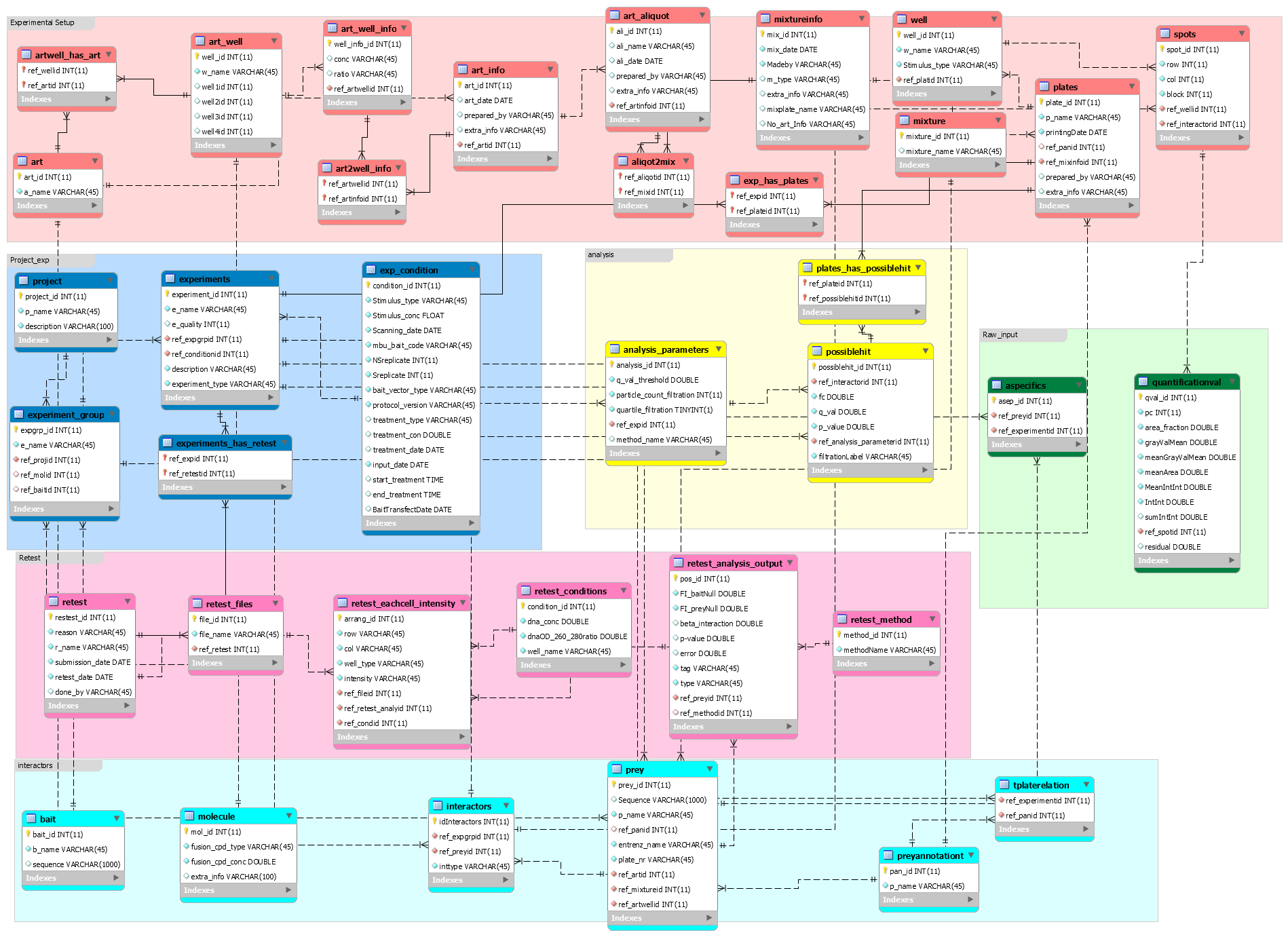


Figure S7 Relational database schema for MAPPI-DAT. Coloured boxes indicate different data types: project and experiment data in dark blue, plate information in red, analysis input data in green, analysis parameters and output data in yellow, retest (validation) data in pink, prey, bait, and molecule data in light blue.

## **References:**

Breitling,R. *et al.* (2004) Rank products: A simple, yet powerful, new method to detect differentially regulated genes in replicated microarray experiments. *FEBS Lett.*, **573**, 83–92.

Heskes,T. *et al.* (2014) A fast algorithm for determining bounds and accurate approximate p -values of the rank product statistic for replicate experiments. *BMC Bioinformatics*, **15**, 367.

Lemmens,I. *et al.* (2010) Strategies towards high-quality binary protein interactome maps. *J. Proteomics*, **73**, 1415–1420.

Lievens,S. *et al.* (2009) Array MAPPIT : High-Throughput Interactome Analysis in Mammalian Cells. 877–886.

Lievens,S. *et al.* (2011) MAPPIT: A protein interaction toolbox built on insights in cytokine receptor signaling. *Cytokine Growth Factor Rev.*, **22**, 321–329.

Lievens,S. *et al.* (2016) Proteome-scale binary interactomics in human cells. *Mol. Cell. Proteomics*, mcp.M116.061994.

Lievens,S. *et al.* (2012) The use of mammalian two-hybrid technologies for high-throughput drug screening. *Methods*, **58**, 335–342.

Storey,J. (2002) A Direct Approach to False Discovery Rates on JSTOR. *Wiley Online Libr.*, **64**, 479--498.

Wolfinger,R.D. *et al.* (2001) Assessing Gene Signi cance from cDNA Microarray Expression Data via Mixed Models. *J. Comput. Biol.*, **8**, 625–637.
